# Supplementary material for: Protective Effects of Sodium Nitroprusside on Photosynthetic Performance of Sorghum bicolor L. under Salt Stress
Source: Plants (Basel). 2023 Feb 13;12(4):832. doi: 10.3390/plants12040832 (PMC9966380; doi:10.3390/plants12040832)
Supplement: Supplementary file 1 [file plants-12-00832-s001.zip › plants-2178728-supplementary.pdf]

Supplementary materials

Table S1. Description of the selected JIP test parameters.

ABS/RC	Absorption flux (of antenna chlorophylls) per reaction center (RC)
ETo/RC	Electron transport flux (further than Q_A^-) per RC
REo/RC	Electron flux reducing end electron acceptors at the PSI acceptor side per RC
TRo/RC	Trapping flux (leading to Q_A reduction) per RC
φ_{Po}	Maximum quantum yield of primary photochemistry (at t = 0)
RC/ABS	Number of Q_A-reducing RCs (i.e., active) per PSII antenna chlorophyll
Vj	Relative variable fluorescence at J step
Wk	Ratio of the K phase to the J phase
PI_{ABS}	Performance index (potential) for energy conservation from exciton to the reduction of intersystem electron acceptors
PItotal	Performance index (potential) for energy conservation from exciton to the reduction of PSI end acceptors
